# The Gut–Brain Axis and Dietary Patterns in Shaping Long-Term Neurocognitive and Psychosocial Outcomes in Adolescent and Young Adult Survivors of Childhood Cancer: A Systematized Narrative Review

**DOI:** 10.3390/nu18172773

**Published:** 2026-08-25

**Authors:** Piotr Pawłowski, Otylia Kościołek, Mikołaj Jeżak, Karol Jakubik, Aneta Kościołek, Marzena Samardakiewicz

**Affiliations:** 1 Department of Psychology, Psychosocial Aspects of Medicine, Faculty of Medicine, Medical University of Lublin, 20-093 Lublin, Poland; 2 Institute of Medical Sciences, University of Applied Sciences in Chełm, 22-100 Chełm, Poland; 3 Student Scientific Club at the Department of Psychology, Faculty of Medicine, Medical University of Lublin, 20-093 Lublin, Poland; 4 I Department of Anaesthesiology and Intensive Care, University Clinical Hospital No. 4 in Lublin, 20-090 Lublin, Poland; 5 Doctoral School, Medical University of Lublin, 20-093 Lublin, Poland; d.karoljakubik@umlub.edu.pl; 6 I Department of Medical Radiology, Medical University of Lublin, 20-090 Lublin, Poland

**Keywords:** childhood cancer survivors, human gut microbiota, neurocognitive functioning, psychosocial functioning, dietary patterns, adolescent and young adult (AYA), long-term follow-up (LTFU)

## Abstract

**Background:** The dynamic advancement of pediatric hemato-oncology and intensified therapeutic protocols have significantly increased survival rates while simultaneously highlighting the challenge of long-term treatment complications. Within the cohort of adolescent and young adult (AYA) survivors, delayed neurocognitive deficits, often manifesting as the chemobrain phenotype, and psychosocial disorders constitute a particularly substantial burden. Contemporary neurogastroenterological evidence indicates a fundamental role of persistent dysbiosis and gut–brain axis dysfunction in the pathogenesis of these alterations. This review aims to critically synthesize translational evidence elucidating the impact of iatrogenic gut microbiota damage and modifiable dietary patterns on the development of long-term neurocognitive sequelae in survivors of early childhood cancer. **Methods:** A systematized narrative review was conducted in accordance with the SANRA guidelines by integrating data from in vivo models and observational studies. A comprehensive literature search across the PubMed, Embase, Cochrane Central, Scopus, and Web of Science databases up to June 2026 was performed utilizing the Population, Exposure, and Outcomes (PEO) framework. **Results:** Oncological therapies, including myeloablative conditioning and broad-spectrum antibiotic therapy, induce a microbial scar phenomenon characterized by the depletion of commensal Firmicutes in favor of resistant pathobionts. The subsequent decline in the synthesis of neuroprotective short-chain fatty acids (SCFAs) alongside the pathological activation of the kynurenine pathway disrupts central nervous system homeostasis. Translocation of lipopolysaccharides (LPSs) across the compromised intestinal barrier generates systemic inflammation recognized as inflammaging, which, in turn, stimulates neurotoxic microglial hyperreactivity. This pathophysiological cascade is accelerated by a pro-inflammatory Western diet, whereas anti-inflammatory interventions such as the MIND diet and postbiotics demonstrate measurable restorative potential. **Conclusions:** The pathophysiology of delayed neurotoxicity is largely a consequence of systemic neuroinflammation driven by intestinal dysbiosis. Implementing individualized dietary and microbiome-targeted strategies into survivorship care protocols constitutes a crucial direction for clinical prophylaxis. Validating their clinical efficacy in the AYA population necessitates prospective randomized controlled trials integrated with shotgun metagenomic sequencing.

## 1. Introduction

The advancement of modern pediatric oncology and hematology has led to the optimization of chemotherapy protocols, the application of precision radiotherapy, and the increasing utilization of targeted and immune therapies. Consequently, there has been a significant increase in cancer survival rates within the pediatric population. In highly developed countries, the five-year survival rate currently exceeds 85%, resulting in a continually growing population of long-term survivors [1].

A novel challenge for healthcare professionals is the phenomenon referred to as the cost of cure. Over the years, the vast majority of childhood cancer survivors (CCSs) experience so-called late effects. It is estimated that even several decades after the completion of intensive antineoplastic therapy, specific conditions and deficits emerge that occur rarely or at a much later stage of life in healthy peers. Among these complications, alongside cardiotoxicity and secondary neoplasms, neurocognitive and psychosocial disorders constitute the most severe problem, substantially impacting the quality of life and the trajectory of holistic development in adolescent survivors [2,3].

Historically, in the literature, the pathogenesis of cognitive impairment was attributed almost exclusively to the direct neurotoxic effects of therapies directed at the central nervous system (CNS). Cranial radiotherapy and the intrathecal administration of cytostatic agents induce demyelination and cerebral white matter loss. Nevertheless, contemporary clinical observations indicate that the phenomenon of cognitive impairment, colloquially referred to as chemobrain or chemofog, also affects patients treated systemically for solid tumors located outside intracranial structures. This phenotype has prompted modern researchers to seek novel pathophysiological mechanisms. A growing body of evidence from the fields of neurogastroenterology, immunology, and metabolomics highlights the critical role of the gut microbiota. Intensive oncological therapies negatively impact the intestinal ecosystem; however, it is crucial to acknowledge that the severity and recovery trajectory of dysbiosis vary dramatically depending on the treatment modality. While standard chemotherapy for solid tumors may provoke more transient shifts in the microbiota, highly intensive protocols, such as myeloablative conditioning prior to hematopoietic stem cell transplantation (HSCT), induce profound and often persistent dysbiosis. Through the gut–brain axis, an impoverished and pathological microbiota generates chronic inflammation and neuroinflammation, thereby disorganizing neurodevelopmental processes. Furthermore, dietary patterns serve as a factor strongly modulating this environment. Dietary habits distorted by the illness experience exacerbate dysbiosis, becoming a significant and importantly modifiable target for therapeutic interventions [2,3,4,5].

Understanding the impact of oncological treatment on the maturing nervous system requires considering the complexity of neurodevelopmental processes occurring in children and adolescents. The vulnerability of the developing nervous system depends on the specific developmental window during which the oncological treatment is administered. Preschool and early school age represent a critical phase for intensive myelination and synaptogenesis, whereas the period of adolescence is characterized by enhanced synaptic pruning within the prefrontal cortex. Early childhood exposure to cytotoxic treatment and the resulting chronic intestinal dysbiosis disrupt these age-specific processes, promoting the phenomenon of so-called sleeper effects. During their course, deficits in higher executive functions do not emerge immediately after the completion of therapy but manifest with a significant delay in early adulthood, when the compensatory capacities of the organism become exhausted in the face of increasing environmental demands [6,7,8,9].

While previous reviews have documented the acute gastrointestinal toxicities of antineoplastic agents or investigated the gut–brain axis primarily in the context of primary neurodegenerative and psychiatric disorders, a gap remains regarding its long-term implications in pediatric oncology. Existing literature frequently focuses on short-term microbiome alterations during active therapy or attributes delayed cognitive decline almost exclusively to the direct neurotoxic impact of chemoradiotherapy. The unique contribution of this systematized narrative review lies in comprehensively mapping the pathophysiological continuum between treatment-induced chronic dysbiosis, systemic neuroinflammation, and the delayed emergence of neurocognitive and psychosocial sequelae, particularly within the vulnerable cohort of adolescent and young adult (AYA) survivors. Furthermore, this review synthesizes translational evidence to evaluate the modulatory potential of targeted dietary interventions, such as the MIND diet, and emerging microbiome-targeted strategies as tangible tools for neuroprotection in long-term survivorship care.

The objective of this paper is to analyze the role of the gut–brain axis and dietary patterns in the development of late neurodevelopmental and psychosocial sequelae of antineoplastic treatment in adolescent and young adult (AYA) survivors of childhood cancer.

## 2. Materials and Methods

### 2.1. Literature Search Strategy

This review was designed as a systematized narrative review integrating a structured approach to evidence identification with the reporting guidelines for narrative reviews, specifically the Scale for the Assessment of Narrative Review Articles (SANRA). To identify relevant preclinical and clinical studies, a comprehensive search was conducted across the following scientific databases: PubMed/MEDLINE, Embase, the Cochrane Library, Scopus, and Web of Science. Additionally, Google Scholar resources were queried to identify gray literature and reports from preprint repositories. These supplementary sources were utilized exclusively to outline a broader context and identify novel experimental research directions rather than serving as a foundation for the primary clinical conclusions.

#### 2.1.1. Timeframe and Language Criteria

Given the dynamic advancement of microbiome sequencing techniques in recent years and the growing awareness of the long-term sequelae of oncological treatment, the search timeframe encompassed publications from January 2010 to June 2026. Only full-text articles published in English or Polish were included in the analysis.

#### 2.1.2. Keywords and Query Construction

The search strategy relied on a combination of controlled vocabulary dictionaries, including Medical Subject Headings (MeSH) and Emtree, along with free-text keywords connected by Boolean logical operators (AND, OR). The primary keyword categories were structured based on the Population, Exposure, and Outcome (PEO) framework, which is highly appropriate for evaluating pathomechanisms and risk factors:

Population: Childhood Cancer Survivors (CCSs) OR pediatric oncology survivors OR adolescent survivors OR young adult survivors OR childhood leukemia OR brain tumors OR hematopoietic stem cell transplantation (HSCT) survivors.

Exposure (gut–brain axis and nutrition): gut-brain axis OR microbiota-gut-brain axis OR gut microbiome OR dysbiosis OR dietary patterns OR Mediterranean diet OR psychobiotics OR probiotics OR nutritional status.

Outcome (neurocognitive and psychosocial sequelae): neurocognitive late effects OR cognitive impairment OR executive functions OR attention deficit OR psychosocial outcomes OR depression OR anxiety OR fatigue OR quality of life (QoL) OR neuroinflammation OR brain-derived neurotrophic factor (BDNF).

An exemplary search query adapted for the PubMed database: (“Childhood cancer survivors” OR “pediatric oncology”) AND (“gut-brain axis” OR “microbiome” OR “dietary patterns”) AND (“neurocognitive” OR “cognitive impairment” OR “psychosocial” OR “depression” OR “quality of life”).

### 2.2. Inclusion and Exclusion Criteria

#### 2.2.1. Inclusion Criteria

The following types of studies were included in the analysis:

(1) Clinical and observational studies: randomized controlled trials (RCTs), long-term cohort studies (both prospective and retrospective), and cross-sectional studies evaluating the population of adolescent (10 to 19 years old) and young adult (up to 25 years of age) survivors of childhood cancer.
(2) Preclinical and experimental studies: animal model studies, such as murine models of chemotherapy-induced cognitive impairment commonly known as chemobrain, providing mechanistic evidence elucidating the impact of the microbiota and its metabolites (e.g., short-chain fatty acids and tryptophan pathways) on neurogenesis, blood–brain barrier integrity, and neuroinflammation.
(3) Review articles and meta-analyses: utilized as reference sources to identify additional key publications via the snowballing approach.

#### 2.2.2. Exclusion Criteria

The following were excluded from the analysis:

(1) Case reports and letters to the editor that did not contribute novel primary data.
(2) Studies focusing exclusively on patients during the acute phase of oncological treatment without an evaluation of long-term sequelae.
(3) Publications lacking full-text availability, conference abstracts, and articles in non-peer-reviewed journals.
(4) Studies lacking validated tools for cognitive function assessment or reliable methods for microbiota and dietary profiling.

### 2.3. Study Selection and Data Extraction Process

#### 2.3.1. Study Selection

The publication selection process was conducted in two independent stages by two researchers:

Initial screening of titles and abstracts to exclude articles irrelevant to the topic of the gut–brain axis in pediatric oncology.

Full-text evaluation of potentially relevant publications for the final confirmation of compliance with the inclusion criteria. Any discrepancies between the two reviewers were resolved through consensus or via arbitration by a third independent researcher. Reasons for the exclusion of full-text records during the secondary screening phase were systematically recorded and classified in accordance with SANRA/PRISMA reporting frameworks (categorized into population ineligibility/lack of long-term follow-up, absence of microbiome/metabolomic markers, lack of validated neurocognitive assessment tools, and ineligible document types).

#### 2.3.2. Data Extraction

The following data were extracted from each eligible study and compiled into structured tables:

Study characteristics: authors, year of publication, study design, and population characteristics including sample size, age at diagnosis, and time since off-therapy.

Oncological data: primary tumor type and applied treatment protocols, with particular emphasis on high-risk factors for neurotoxicity such as central nervous system radiotherapy, intrathecal chemotherapy, and high-dose methotrexate or cytarabine.

Microbiome and dietary assessment methods: taxonomic resolution of microbiota analysis, types of dietary questionnaires, and biochemical marker concentrations.

Neurocognitive and psychosocial assessment methods: a registry of administered test batteries, quality of life evaluation (e.g., PedsQL), and diagnosis of potential psychiatric disorders according to DSM-5 criteria.

Main results: identified dysbiosis patterns, such as a decrease in alpha diversity or depletion of commensal bacteria, their correlation with cognitive deficits and anxiety or depressive states, alongside the modifying role of specific dietary patterns.

### 2.4. Data Synthesis Methods

Due to the translational nature of this paper, which involves integrating preclinical evidence from in vivo models with data from observational clinical studies characterized by high methodological heterogeneity, conducting a formal meta-analysis was waived. A narrative synthesis approach was employed, focusing on the following areas:

Identifying pathognomonic patterns of post-treatment dysbiosis, known as the microbial scar, in adolescents and correlating them with delayed neurotoxicity.

Critically analyzing the impact of pro-inflammatory and neuroactive microbiota metabolic pathways, including the tryptophan to kynurenine axis and short-chain fatty acid production, on the central nervous system integrity in post-antineoplastic therapy patients.

Evaluating the modulatory potential of microbiota-targeted interventions, such as individualized dietary patterns and psychobiotic supplementation, in providing psychosocial support and improving executive functions.

The results were categorized and are presented in clear thematic subsections corresponding to the pathophysiological logic of the gut–brain axis.

## 3. Results

**Figure 1:**
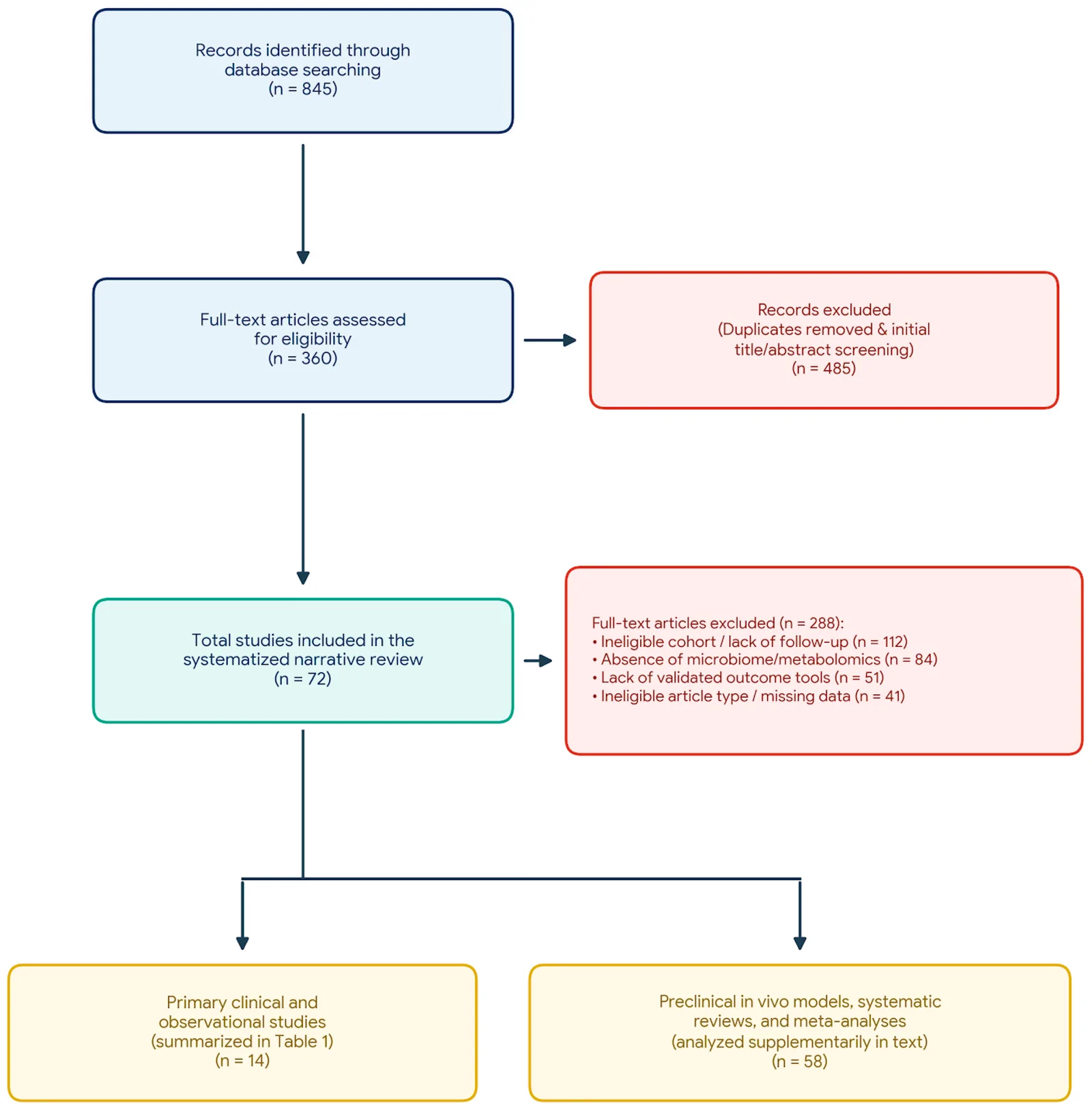
Flow diagram illustrating the identification, screening, eligibility, and inclusion process for the systematized narrative review in accordance with SANRA/PRISMA standards. A total of 288 full-text articles were excluded with specified reasons: ineligible cohort or lack of long-term survivorship follow-up (*n* = 112), absence of gut microbiome or gut–brain axis metabolomic metrics (*n* = 84), lack of validated neurocognitive/psychosocial outcome assessments (*n* = 51), and ineligible article type or missing primary data (*n* = 41). The 72 included publications comprise 14 primary clinical and observational studies (summarized in Table 1) and 58 non-clinical translational evidence sources (preclinical in vivo models, reviews, systematic reviews, and meta-analyses analyzed supplementarily in text).

The comprehensive literature search across the designated databases yielded an initial total of 845 records. Following the removal of duplicates and initial title/abstract screening (*n* = 485), 360 full-text articles were rigorously evaluated for eligibility. Of these, 288 full-text articles were excluded based on predefined criteria for the following primary reasons: ineligible study population or lack of long-term survivorship follow-up (*n* = 112), absence of gut microbiome profiling or gut–brain axis metabolomic metrics (*n* = 84), lack of standardized/validated neurocognitive or psychosocial outcome assessments (*n* = 51), and ineligible publication type or inaccessible primary data (*n* = 41). Ultimately, 72 publications met all eligibility criteria and were synthesized in this systematized narrative review (Figure 1).

The structural and methodological characteristics of these core clinical and observational studies, including population specifics, exposure/outcome assessment methods, and primary findings, are summarized in Table 1. Preclinical in vivo models utilized to elucidate specific molecular mechanisms of the gut–brain axis were analyzed supplementarily in the text.

### 3.1. Cognitive Impairment During Antineoplastic Treatment in the Pediatric Population

Studies demonstrate that approximately 50% to 60% of children treated for malignancies bear a significant risk of developing neurocognitive impairments. Although this issue is most pronounced in patients subjected to cranial irradiation at a young age, who exhibit a decline in intelligence quotient of up to 20 to 50 points, these dysfunctions affect a broad group of survivors [19,21,22]. Among adolescents and young adults in the survivorship phase, difficulties are most frequently observed in domains such as attention, working memory, information processing speed, and higher executive functions including planning, cognitive flexibility, and response inhibition [4]. An analysis conducted on a cohort of 1106 young adult survivors revealed that overall, 13% of the population reports severe learning and memory problems [14].

The high prevalence of these deficits applies not only to individuals following the treatment of acute lymphoblastic leukemia (ALL) or brain tumors but also to patients diagnosed with solid tumors. Studies on long-term outcomes in Ewing sarcoma and osteosarcoma survivors demonstrated that nearly 15 years post-diagnosis, at a median age of merely 28 years, 34.5% suffer from processing speed deficits, 18.1% from cognitive flexibility impairments, and up to 13% exhibit significant concentration problems [11]. Furthermore, similar difficulties emerge in patients treated for Wilms tumor. One study revealed that they achieved significantly lower scores than the healthy population across most cognitive variables, and the rate of memory and attention disorders increased linearly with the presence of other neurological and somatic comorbidities [1].

### 3.2. Psychosocial Dimensions of Late Effects in Childhood Cancer

Cognitive dysfunctions co-occur with psychosocial and emotional problems, forming a construct known as psychoneurological symptoms. This syndrome encompasses mutually perpetuating disorders such as neuropathic pain, chronic fatigue, insomnia, anxiety, and profound depressive symptoms [7]. Social withdrawal and difficulties in maintaining peer relationships manifest with a delay, often presenting during adolescence when the expectations of the academic and professional environment increase [4]. Survivors exhibit significantly higher rates of poor mental health compared to the general population, with a particular emphasis on lowered self-esteem and a sense of alienation [15].

Executive function impairments and emotional disorders strongly translate into socioeconomic capabilities. A lower level of education is noticeably prevalent. In one study, Wilms tumor survivors exhibited a more than twofold higher risk of failing to complete higher education, marked by an odds ratio of 2.23 [1]. These issues directly impact professional functioning. Research on a cohort of sarcoma patients revealed that individuals compelled to work more than nine hours daily presented significantly poorer parameters of attention and cognitive flexibility, indicating a rapid depletion of their neurocompensatory reserves within the stressful environment of adulthood [11]. Consequently, this leads to the phenomenon of financial toxicity. Survivors struggle with funding essential, comprehensive healthcare, while neurocognitive deficits hinder their ability to effectively navigate insurance systems, for example, in the United States. Economic and social stress additionally burdens the hormonal hypothalamic–pituitary–adrenal axis, negatively affecting mood and overall functioning [20].

Adolescents affected by cancer also face the problem of behavioral coping mechanisms in response to the stress resulting from the chemobrain phenomenon. Analyses demonstrate that self-reported cognitive impairment in young adult childhood cancer survivors correlates with a statistically higher risk of resorting to substance use. Among others, a more than twofold increased risk of regular e-cigarette use, or vaping, was identified in individuals with cognitive deficits, presenting an odds ratio of 2.26. Consequently, cognitive impairments exacerbate addictive behaviors, serving as a maladaptive attempt to self-medicate chronic stress, fatigue, and depression [14]. Table 2 presents the characteristics of long-term psychosocial and cognitive sequelae and their dependence on the type of neoplasm.

### 3.3. Gut Microbiota and Oncological Treatment

A healthy, physiologically functioning pediatric gastrointestinal tract is colonized by trillions of commensal microorganisms whose diversity and equilibrium, known as eubiosis, are crucial for the proper maturation of metabolic, immune, and neurological functions [23]. Microbiome development is a dynamic process dependent on the mode of delivery, breastfeeding, diet, and early environmental exposures. It is worth emphasizing that the moment of cancer diagnosis and the initiation of adequate therapy negatively impact this ecosystem. Furthermore, it has been demonstrated that exposure to microbes, including commensal ones, during early life stages modulates immunity, which may even serve as a protective factor against childhood leukemias [24].

Pediatric oncological treatment relies on intensive strategies. Aggressive chemotherapy protocols targeting rapidly dividing cells cause the destruction of not only the tumor mass but also intestinal epithelial cells, inducing severe mucosal inflammation termed mucositis. The destruction of the mucin layer and the sloughing of intestinal villi alter the physicochemical properties of the intestinal lumen. Physiologically, this environment is strictly anaerobic, which favors the growth of health-critical bacteria from the *Firmicutes* and *Bacteroidetes phyla* [25]. Inflammation and mucosal bleeding introduce oxygenated blood into the intestinal lumen, promoting the proliferation of facultative anaerobic pathobionts, particularly from the *Proteobacteria phylum*, including pathogens such as *Klebsiella* or *Escherichia coli* species. Additionally, for prophylactic purposes or the treatment of neutropenic fever, pediatric oncology patients are routinely subjected to intensive broad-spectrum antibiotic therapy. This results in the eradication of beneficial strains, including those from the *Bifidobacteriumand Faecalibacterium* genera, which produce protective short-chain fatty acids that maintain the integrity of the intestinal barrier [26].

In the context of hematopoietic malignancies, particularly in patients undergoing allogeneic hematopoietic stem cell transplantation, the degree of intestinal ecosystem devastation reaches an extreme form [16]. The application of myeloablative conditioning, relying, among other things, on the administration of high doses of cyclophosphamide and total body irradiation, induces massive apoptosis of intestinal crypt epithelial cells, disrupting mucosal architecture and destroying the niche for commensal bacteria [27]. In translational analysis, it is essential to clearly differentiate the dynamics of dysbiosis induced by specific protocols. While standard chemotherapy for solid tumors typically provokes a transient reduction in biodiversity, total body irradiation-based myeloablative conditioning and prolonged empirical broad-spectrum antibiotic therapy, which are indispensable in managing neutropenic fevers in hematology patients, impact the microbiome to an extreme degree [16,28]. This generates a profound and frequently persistent microbial scar, characterized by the irreversible loss of species from the *Firmicutes phylum* in favor of multidrug-resistant pathobionts from the *Proteobacteria phylum* [18,28]. This treatment-specific baseline heterogeneity determines varying intensities of systemic inflammation and directly translates into a differentiated risk of secondary gut–brain axis neuroinflammation. An additional critical challenge within this patient group is the occurrence of graft-versus-host disease affecting the gastrointestinal tract. This donor lymphocyte-dependent immune response constitutes a potent, independent factor damaging the mucosa and inducing a cytokine storm [29]. Pro-inflammatory cytokines released locally in massive quantities enter the bloodstream and subsequently freely cross the compromised blood–brain barrier, exacerbating central neuroinflammation [30]. This mechanism establishes a direct, systemic pathophysiological cascade linking hematological treatment toxicity with progressive neurocognitive impairments [30,31].

A key discovery explaining the development of late effects in the adolescent survivor population is the fact that the restoration of clinical health is not synonymous with the complete reconstitution of the gut microbiota. However, the magnitude and permanence of this ‘microbial scar’ are inextricably linked to the severity of the initial therapeutic insult. While survivors of standard solid tumor chemotherapy may demonstrate partial microbial recovery over time, those subjected to myeloablative conditioning (HSCT/TBI) face a vastly different and more severe dysbiotic baseline [28]. Consequently, even a decade after the completion of such intensive antineoplastic therapy, these specific patient subgroups exhibit a profoundly entrenched dysbiotic signature, referred to as late-onset dysbiosis. Genetic analyses based on 16S rRNA gene sequencing have demonstrated that alpha diversity, denoting species richness, and beta diversity, reflecting microbial community structure, remain drastically altered compared to healthy siblings and the general control population [3].

Studies conducted among young adult survivors of ALL, with a median age of 26 years and a post-treatment interval of 18.5 years, confirmed the sustained depletion of protective *Faecalibacterium* species alongside an unnatural overgrowth of *Actinobacteria* strains, including *Corynebacterium* [12]. This dysbiosis is not strictly localized. Deficits in the commensal structure correlate with elevated concentrations of plasma inflammatory markers, such as interleukin-6 and C-reactive protein, as well as the presence of activated T lymphocyte populations, specifically CD4+ and CD8+ cells expressing HLA-DR [3]. The translocation of bacterial antigens and lipopolysaccharides across a permanently damaged, leaky intestinal barrier leads to metabolic endotoxemia [32]. It is precisely this chronic inflammatory state, recognized as inflammaging, that is considered the foundation for the development of metabolic syndrome, visceral obesity, and progressive central nervous system pathologies among childhood cancer survivors [25].

### 3.4. The Gut–Brain Axis in the Development of Chemobrain: Ascending and Descending Signals

The brain and the gastrointestinal tract maintain a constant, dynamic connection. In its functioning, the gut–brain axis utilizes diverse communication channels encompassing neural, immunological, hormonal, and metabolic pathways [33]. In adolescent survivors, the dysregulation of this axis is therefore of critical importance in shaping the local toxicity of chemotherapy and its subsequent central complications. Current reports indicate that drugs with a low capacity to cross the blood–brain barrier can induce profound cognitive deficits, particularly in a highly vulnerable cohort such as the pediatric population [34].

In preclinical models, intestinal inflammation resulting from mucositis and pathogen overgrowth generates a surge of pro-inflammatory cytokines, predominantly IL-1β and TNF-α. Experimental data show that these compounds enter the systemic circulation and reach the brain, where they can infiltrate neural tissue directly via the circumventricular organs or induce dysfunction in the tight junction proteins of the blood–brain barrier itself [34]. Consequently, in these in vivo paradigms, when inflammatory signals and endotoxins reach the brain parenchyma, they trigger the activation of microglia and astrocytes. When inflammatory signals, accompanied by bacteria and their endotoxins, reach the brain parenchyma, they trigger the activation of microglia and astrocytes. Microglia, which physiologically fulfill a protective role, transition into a neurotoxic state, destroying surrounding neurons and synapses, particularly in critical learning regions such as the hippocampus. These cells also undergo a phenomenon known as glial priming. This implies that even long after the cessation of chemotherapy, microglia remain in a state of hyperreactivity, responding with a destructive inflammatory cascade to everyday stressors [34]. The majority of mechanistic evidence confirming the involvement of hyperreactive microglia in the pathogenesis of chemobrain originates from preclinical in vivo models, where the activation of these cells strongly correlates with behavioral impairments in spatial orientation tests [35]. However, extrapolating these phenomena to the human population requires substantial caution. In oncological patients within the childhood cancer survivor group, dysfunctions in the higher echelons of the gut–brain axis are primarily diagnosed indirectly. They are based on the correlation between elevated titers of peripheral inflammatory biomarkers, such as IL-6, C-reactive protein, and lipopolysaccharide endotoxemia, and volumetric changes in white matter documented in neuroimaging studies, along with a decline in performance on standardized psychometric tests [36]. A research team from the Wroclaw Medical University confirmed that even trace amounts of circulating endotoxins disrupt microglial function and damage microvessels through the release of deleterious exosomes and specific microRNAs, serving as a prelude to premature brain aging and dementia [37].

The vagus nerve constitutes the primary neural connection between the abdominal cavity and the brainstem. Nearly 90% of its fibers serve sensory purposes, informing the brain about the intestinal environment. Vagal receptors exhibit the competence to detect alterations in the release of gut hormones and the presence of bacterial metabolites. Inflammatory and stress signals from the viscera rapidly reach the nucleus of the solitary tract and are subsequently transmitted to other central structures, including the limbic system, prefrontal cortex, and amygdala, thereby shaping behavioral responses, modulating anxiety levels, and generating the visceral pain typical of oncological patients [34].

Preclinical evidence indicates that microbiome depletion in survivors deprives the central nervous system of key regulatory molecules produced almost exclusively in the intestinal lumen [5]. Butyrate, acetate, and propionate, generated through the fermentation of dietary fiber by Bifidobacterium and Firmicutes, possess the unique ability to cross the blood–brain barrier. Their role involves modulating neuroplasticity, activating the synthesis of brain-derived neurotrophic factor, and reinforcing the integrity of the vascular endothelium within the brain [5]. A low titer of short-chain fatty acids represents a significant factor in memory impairment and the exacerbation of psychoneurological pathologies [26]. In a healthy individual, tryptophan is the precursor to serotonin, which is essential for mood regulation. Observational human studies suggest that following chemotherapy, an overgrowth of specific pathobionts, including *Intestinibacter* and *Megasphaera*, occurs, which preferentially shifts the tryptophan pathway toward kynurenine in a pro-inflammatory environment. Kynurenine crosses the blood–brain barrier and is converted within the brain into neurotoxic quinolinic acid, an NMDA receptor agonist, resulting in neuronal apoptosis and the generation of profound depressive and anxiety symptoms [7]. These phenomena are critical considering that over 90% of all human serotonin is produced with the involvement of the intestinal microbiome, for example, by *Enterococcus* strains [38]. A reduced population of bacteria from the *Lactobacillus* and *Bifidobacteriumgenera* decreases the pool of bacterial gamma-aminobutyric acid, the principal inhibitory neurotransmitter in the central nervous system, the deficiency of which manifests as intensified anxiety and restlessness [39].

The pathway from the brain to the gut is equally critical. Post-traumatic stress disorder related to oncological treatment, the loss of peers, constant fear of disease recurrence, and struggles with neurocognitive dysfunctions activate the sympathetic nervous system and the hypothalamic–pituitary–adrenal axis. This stress induces a descending release of cortisol and catecholamines, particularly adrenaline [14,20]. These compounds inhibit gastrointestinal motility, reduce the production of protective mucus, and directly promote the proliferation of virulent pathogenic bacteria within the intestinal lumen. Consequently, the psychological reaction to the disease perpetuates organic dysbiosis, creating a feedback loop that is exceedingly difficult to interrupt [34].

**Figure 2:**
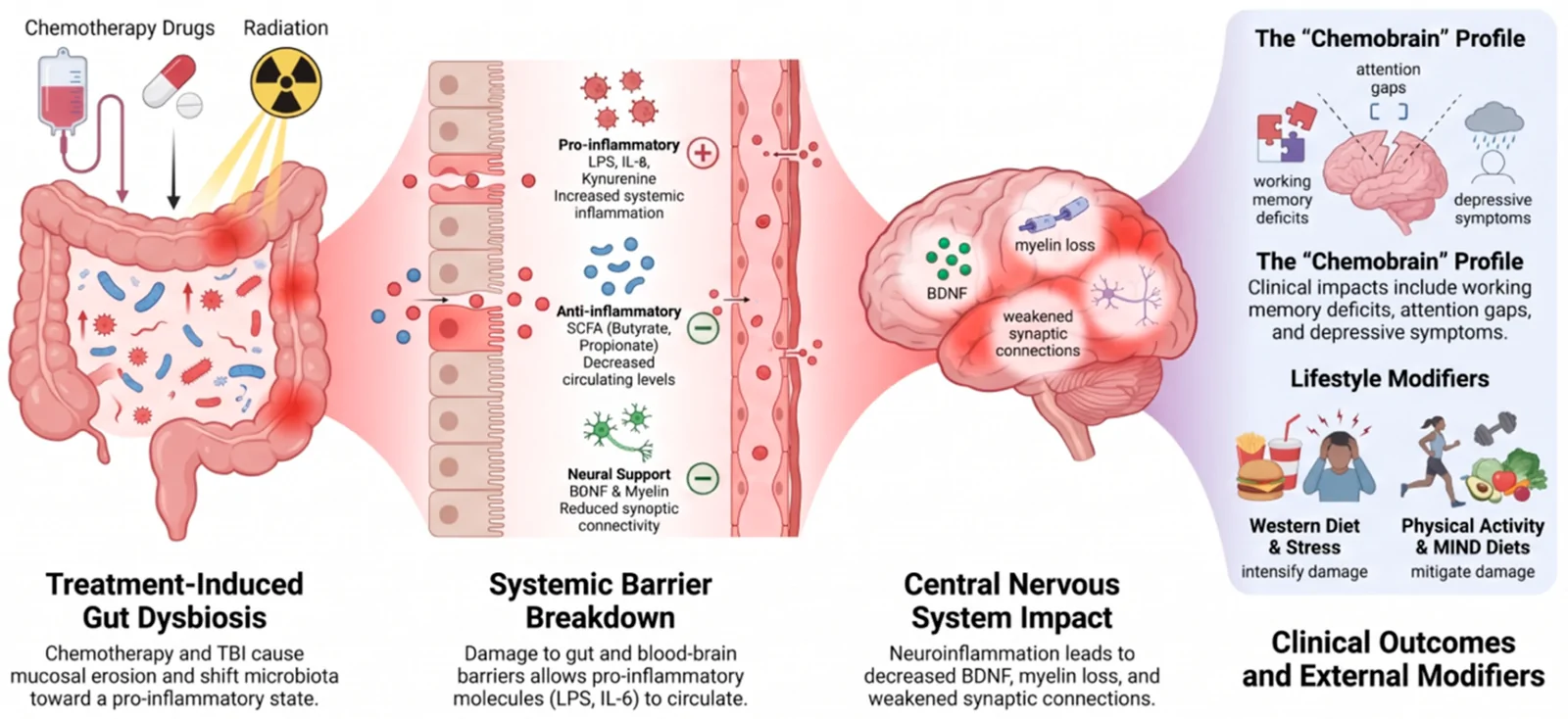
Communication pathways of the gut–brain axis and neurocognitive and psychosocial symptoms in childhood cancer survivors. Abbreviations: BDNF, brain-derived neurotrophic factor; IL, interleukin (e.g., IL-6, IL-8); LPS, lipopolysaccharide; MIND, Mediterranean-DASH Intervention for Neurodegenerative Delay; SCFA, short-chain fatty acids; TBI, total body irradiation.

For clarity, the mechanistic biological pathways bridging the gut–brain axis are visualized in Figure 2. Correspondingly, the clinical translation of how these specific disruptions manifest as neurocognitive and psychosocial symptoms in survivors is systematically summarized in Table 3.

A critical evaluation of the clinical evidence linking dysbiosis with cognitive deficits in the AYA population requires the consideration of significant confounding variables. The state of the microbiome and the ultimate functional capacity of the nervous system are strongly determined by genetic factors (e.g., BDNF gene polymorphisms or the APOE ε4 allele), the widespread use of antibiotic therapy for typical community-acquired infections during the survivorship phase, body mass index fluctuations, and the heterogeneous socioeconomic status of the families [17,40,41,42,43]. Furthermore, post-treatment lifestyle behaviors—particularly physical inactivity and the use of substances such as e-cigarettes or conventional tobacco-alongside the concurrent administration of psychotropic medications for emerging mental health disorders, must be strongly emphasized as critical covariates. These factors possess the independent capacity to profoundly modulate the intestinal microbial architecture and simultaneously influence cognitive performance scores, thereby complicating the isolation of primary, treatment-specific effects. Current literature relies predominantly on observational cross-sectional studies of an associative nature. Defining unequivocal causal cascades between the phenotype of the post-radiation or post-chemotherapy microbiome and the phenomenon of delayed neurotoxicity will necessitate the future design of prospective longitudinal studies rigorously controlling for the aforementioned covariates.

### 3.5. Dietary Patterns in Survivors and Neurocognitive and Psychosocial Disorders

In light of the evidence connecting microbiome alterations with a decline in nervous system performance, daily diet and lifestyle modifications emerge as an important extrinsic instrument for prevention. Understanding how distorted dietary patterns exacerbate damage along the gut–brain axis can form the foundation for modern long-term care [44].

The challenging experiences endured during oncological treatment leave indelible marks on a child’s psyche. Nausea, vomiting, and taste perception disorders, known as dysgeusia, which are prevalent in pediatric patients and generated by chemotherapy and severe oral mucositis, lead to a phenomenon termed learned food aversions [13,45]. Food, which under physiological conditions should provide pleasure, frequently becomes a source of pain for the oncological patient. Furthermore, parental anxiety regarding the child’s malnutrition and cachexia fosters permissiveness toward the consumption of calorie-dense and nutritionally unbalanced foods. This initiates detrimental patterns of emotional overeating and the phenomenon of picky eating, which typically persist relatively long after the conclusion of hospitalization [46].

Survivors generally enter adolescence with an established and extremely unfavorable dietary profile [46,47]. Study results demonstrate that young childhood cancer survivors consume significantly more so-called empty calories in the form of highly processed foods, refined sugars, and saturated fats, while simultaneously avoiding fruits, vegetables, and fiber sources when compared to the control group. It is estimated that in over half of the observed survivors, the energy intake drastically exceeds the requirements without meeting the minimal demand for microelements and vitamins [46,47].

The aforementioned dietary pattern, resembling the Western diet, exacerbates ongoing dysbiosis through the elimination of health-promoting microbes, such as the starvation of cellulolytic strains, and the predominance of pro-inflammatory bacteria. This also intensifies the phenomenon of visceral obesity, insulin resistance, and premature cardiovascular diseases [3,48,49]. Regarding the chemobrain phenomenon, it has been demonstrated that patients who develop metabolic syndrome with chronic overweight achieve substantially poorer results in attention and executive function tests compared to patients with a normal body weight [3,50]. Chronic tissue hypoxia, oxidative stress induced by a hyperglycemic state, and circulating adiponectin from dysfunctional adipose tissue act synergistically with lipopolysaccharide translocating from the gut, thereby exacerbating damage to the cerebral white and gray matter. Additionally, low physical activity, which reduces cerebral blood flow, constitutes the strongest independent predictor of attention deterioration in childhood cancer survivors [44]. Crucially, when interpreting these associations, the potential for reverse causality must be explicitly acknowledged within this observational framework. It is highly plausible that primary, treatment-induced executive dysfunctions and concurrent depressive symptoms impair self-regulation and drive the behavioral preference for a highly processed Western diet. In this scenario, poor diet quality may be a secondary consequence of early neurocognitive deficits rather than the sole primary initiator of cognitive decline, ultimately creating a mutually perpetuating feedback loop.

Redirecting the habits of survivors toward patterns based on the Mediterranean diet or the CNS-dedicated MIND diet (Mediterranean-DASH Intervention for Neurodegenerative Delay) yields promising theoretical effects by preventing complications at the cellular level [51,52,53,54]; however, high-quality interventional data in the pediatric oncology cohort remain limited.

The consumption of large quantities of prebiotic fiber significantly improves the ratio of beneficial *Bacteroidetes* to pathogenic *Proteobacteria*. In preclinical model studies of childhood stress, the application of a high-cellulose diet reduced cell destruction by over 33%, evidenced by a decreased population of Iba-1 positive microglia in the hippocampus, and led to a 30% improvement in object localization capabilities. Fecal microbiota transplantation from mice adhering to this diet also resulted in a cognitive function improvement of up to 46%. This evidences the direct, restorative effect of fiber on the communication pathways within the gut–brain axis [55].

Natural pigments from berries, alongside polyphenols from green tea and seeds, act similarly to prebiotics. They selectively promote the growth of *Bifidobacterium* while simultaneously inhibiting the proliferation of pathogenic microorganisms. They also attenuate oxidative stress, which is detrimental to the intestinal microbiome. The metabolites of these compounds, such as protocatechuic acid, penetrate the central nervous system, significantly reducing the state of neuroinflammation [56,57,58].

Essential polyunsaturated fatty acids, including EPA and DHA, constitute a vital element in the reconstruction of cell membranes and myelin sheaths destroyed by radiotherapeutic and chemotherapeutic interventions. Supported by the B-vitamin complex, they reduce brain fog, alleviate fatigue, facilitate more efficient synaptic communication, and lower the risk of chronic depression occurrence [59,60,61,62]. Table 4 presents proposed dietary interventions demonstrating benefits for the intestinal microbiome as well as the neurocognitive and psychosocial health of survivors.

### 3.6. Therapeutic Strategies Based on the Microbiome and Health Promotion

The discovery of the relationship between diminished microbiome diversity and the pathophysiology of neuropsychiatric complications has opened novel preventive avenues for young oncological patients. Interventions targeting the gastrointestinal tract prove to be less invasive and concurrently often more direct than attempts to treat neurodevelopmental consequences [63,64].

The utilization of live bacterial cultures with a clinically documented impact on the psychological sphere, referred to as psychobiotics, raises significant hopes among researchers. Supplementation with selected strains, predominantly from the Lactobacillus and *Bifidobacterium* genera, aims to competitively exclude pathogenic microbes while simultaneously supplying the nervous system with precursors for neurotransmitters such as GABA [65,66]. Animal models have demonstrated that the daily and early administration of probiotics from the initiation of treatment with cardiotoxic and neurotoxic doxorubicin until adulthood prevented the development of chronic dysbiosis and subsequently abolished chemotherapy-induced memory impairments and anxiety. Protective strains enhanced hippocampal neurogenesis and preserved the full expression of synaptic proteins [35,67]. The application of comprehensive solutions known as synbiotics, which contain both bacteria and carbohydrates serving as prebiotics, significantly accelerates this process, facilitating intestinal colonization by lost commensals [25,68].

Crucially, due to profound and often prolonged immunodeficiencies, particularly in patients following hematopoietic stem cell transplantation (HSCT) or those experiencing prolonged neutropenia the administration of live bacterial strains carries a well-documented risk of opportunistic bacteremia and fungemia. Consequently, the routine use of live psychobiotic biotherapeutics in actively immunocompromised survivors is generally contraindicated. Therefore, the attention of oncologists is prudently shifting toward postbiotics, which encompass ready-made bacterial metabolites, isolated cell membrane fragments, or inactivated cells. Their administration supplies neuroprotective short-chain fatty acids and immunomodulators without introducing live organisms into a sterilized body, providing a safe alternative that protects the central nervous system and mucosa against the effects of cytostatic agents [5,66]. However, it must be critically noted that the current state of knowledge regarding the efficacy of psychobiotics in pediatric oncology relies predominantly on inferences from animal models and the extrapolation of results from other clinical populations, such as patients with autism spectrum disorder or irritable bowel syndrome. To date, there is a lack of multicenter randomized placebo-controlled trials that would unequivocally prove the direct efficacy and long-term safety of targeted strain supplementation in preventing neurocognitive complications in cured patients.

In cases of severely devastated intestinal flora, fecal microbiota transplantation from selected healthy donors remains a promising solution. Although the efficacy of fecal microbiota transplantation (FMT) has been demonstrated in patients with primary neurological disorders, its application in pediatric oncology remains strictly limited to highly controlled clinical trials. This restriction is dictated by a critical safety caveat: the severe risk of transmitting multidrug-resistant pathogens or undetected opportunistic viruses to an immunocompromised host. Nevertheless, provided that rigorous donor screening protocols and advanced filtering techniques are perfected, it exhibits enormous potential for the cognitive and physiological restoration of microbiome function, particularly in patients following total body irradiation [25,69,70].

Isolated dietary modifications are frequently insufficient without behavioral support. Comprehensive health promotion initiatives engaging young patients and their families in exercise regimens and nutritional education represent a key direction in the development of prophylactic therapies. Low physical activity is associated with a decrease in gray matter volume, a greater decline in attentional executive functions, and an exacerbation of the frailty phenomenon in young adult survivors. Moderate-intensity aerobic physical exercise constitutes an independent factor supporting the microbiome by stimulating butyrate production and facilitating the regulation of glycemia and intestinal transit motility, which in adolescents can reduce oxidative stress and alleviate feelings of chronic fatigue [10,44,71,72].

The application of modern methods, such as mobile applications for monitoring the intake of fruits and flavonoids and establishing precise training regimens including a minimum specified weekly exercise duration, can yield significantly higher efficacy and adherence in adolescents than passive in-office counseling [25].

## 4. Study Limitations

This systematized narrative review possesses specific limitations that should be considered when interpreting the formulated conclusions. These arise primarily from the adopted review methodology and the nature of the included primary studies.

Despite the application of a structured search strategy in accordance with SANRA guidelines, this paper does not constitute a systematic review with meta-analysis. The extreme heterogeneity of the included studies, encompassing diverse oncological protocols, distinct microbiome profiling methods, and various neurocognitive test batteries, precluded mathematical data synthesis, such as calculating a pooled effect for individual dysbiosis indicators, and a standardized risk of bias assessment.

Restricting the search exclusively to English and Polish publications might have resulted in the omission of significant reports from other linguistic regions, posing a risk of publication bias.

A substantial portion of the mechanistic evidence confirming the communication pathways of the gut–brain axis, such as microglia-induced neuroinflammation and the impact of dietary fiber, relies on preclinical in vivo models. Fundamental interspecies differences exist in gastrointestinal architecture, commensal profiles, and central nervous system maturation dynamics between rodents and humans, necessitating caution in the direct extrapolation of these mechanisms to the adolescent and young adult population.

The majority of the included clinical studies involving human survivors are cross-sectional or retrospective in nature. Such a study design only permits the demonstration of associations rather than robust causal relationships. Currently, the phenomenon of reverse causality cannot be unequivocally excluded, representing a scenario where primary post-radiation neurocognitive deficits and developing depression in adolescents determine the choice of a highly processed Western diet, which only secondarily drives intestinal dysbiosis.

Many of the analyzed papers relied on 16S rRNA gene amplicon sequencing, which typically allows for microbiota identification down to the genus level. This tool does not provide the functional resolution offered by shotgun metagenomic sequencing, which limits the possibilities for precisely mapping metabolic pathways, such as the capacity of specific strains to produce short-chain fatty acids or modulate the tryptophan pathway, in oncological patients.

The widespread use of food frequency questionnaires in observational studies among adolescents carries a high risk of recall bias and social desirability bias, which may lead to the underestimation of highly processed food intake and confound the correlations between diet and cognitive functions.

## 5. Perspectives and Future Research Directions

The verification of mechanistic hypotheses, currently relying predominantly on preclinical studies and cross-sectional observations, requires the design of multicenter prospective longitudinal randomized controlled trials. Within these trials, transitioning from basic taxonomic profiling to metagenomic sequencing is essential. Only the functional resolution offered by this technology will enable the precise mapping of metabolic pathways, including the enzymatic capacity of the microbiota for short-chain fatty acid synthesis or tryptophan conversion, in oncological patients over the years. Furthermore, future research protocols should integrate advanced microbiome diagnostics with the monitoring of targeted intervention safety and efficacy. Considering the risk of infectious complications in this specific cohort, research on postbiotics should become a focal point. Evaluating the implementation of safe, isolated bacterial metabolites in survivors navigating the adaptively challenging period of adolescence currently constitutes a priority in the pursuit of minimizing the delayed neurotoxicity of oncological treatment.

## 6. Conclusions

The therapeutic success in pediatric oncology survival rates necessitates a fundamental paradigm shift in long-term survivorship care. Rather than viewing late neurocognitive and psychosocial sequelae as inevitable, isolated consequences of central nervous system (CNS) toxicity, current evidence underscores the crucial mediating role of the gut–brain axis. Treatment-induced “microbial scarring” and subsequent chronic systemic neuroinflammation constitute a critical pathophysiological link in the development of the post-chemotherapy brain phenotype in adolescent and young adult survivors.

Consequently, standard clinical follow-up protocols must evolve to adopt a more holistic, multisystemic approach. The integration of microbiome-targeted strategies offers a novel, actionable framework for neuroprotection. Ultimately, redefining survivorship care to incorporate active surveillance and rehabilitation of the gut ecosystem will be instrumental in mitigating delayed neurotoxicity, safeguarding long-term cognitive trajectories, and restoring overall psychosocial well-being in the expanding population of childhood cancer survivors.

## Figures and Tables

**Table 1:** Summary characteristics of the key clinical and observational studies included in this review.

Author & Year	Study Design	Population & Cancer Type	Sample Size (N)	Exposure Assessment (e.g., Microbiome/Diet)	Outcome Assessment (Neurocognitive/Psychosocial)	Main Findings
Bielik et al., 2023 [10]	Clinical intervention/observational study	Childhood cancer survivors	*N* = 32 (16 patients vs. 16 controls)	Physical exercise program and dairy probiotics (*Lactobacillus casei*)	Gut microbiome diversity, SCFA production	Demonstrated that combining moderate physical exercise with specific probiotic supplementation positively modulated the gut microbiome and stimulated protective butyrate production [10].
Cheung et al., 2023 [11]	Cross-sectional observational study	Chinese AYA survivors of sarcoma	*N* = 116	Lifestyle factors (physical activity, daily working hours)	Neurocognitive impairment (attention, processing speed, cognitive flexibility)	34.5% exhibited processing speed deficits; low physical activity and working >9 h/day significantly exacerbated the depletion of neurocompensatory reserves [11].
Chua et al., 2020 [12]	Longitudinal observational study	Children with Acute Lymphoblastic Leukemia (ALL)	*N* = 14 (7 patients vs. 7 controls)	Chemotherapy timeline (prior, during, and post-cessation)	Temporal changes in gut microbiota profile (16S rRNA)	Confirmed sustained depletion of commensal strains and unnatural overgrowth of *Actinobacteria* (e.g., *Corynebacterium*) persisting long after therapy cessation [12].
Cohen et al., 2021 [13]	Observational cross-sectional study	Children actively undergoing cancer treatment	*N* = 36	Dietary intake (food records and nutritional tracking)	Diet quality, early nutritional status	Revealed that pediatric patients rapidly develop poor diet quality during active therapy, establishing detrimental eating patterns that persist into the survivorship phase [13].
Ng et al., 2023 [14]	Retrospective cohort analysis (Project Forward)	Young adult survivors of childhood cancer	*N* = 1106	Self-reported cognitive impairment status	Substance use prevalence (e.g., e-cigarettes/vaping)	13% reported severe memory problems; cognitive impairment correlated with a >2-fold increased risk of vaping as a maladaptive coping mechanism (OR = 2.26) [14].
O’Connor, 2023 [15]	Clinical cohort analysis	Pediatric cancer survivors	*N* > 3.1 million (GWAS data context)	History of childhood cancer treatment	Mental health disorders prevalence	Survivors exhibit significantly higher rates of poor mental health compared to the general population, marked by lowered self-esteem and alienation [15].
Olsson et al., 2019 [1]	Retrospective cohort study (St. Jude Lifetime Cohort)	Long-term survivors of Wilms tumor	*N* = 158 survivors (vs. *N* = 354 controls)	History of solid tumor antineoplastic treatment	Neurocognitive testing (memory, verbal fluency, mathematics)	Survivors achieved significantly lower cognitive scores than healthy peers; cognitive impairment correlated with a >2-fold higher risk of failing to complete higher education (OR = 2.23) [1].
Peled et al., 2020 [16]	Clinical observational cohort study	Patients undergoing allogeneic hematopoietic cell transplantation	*N* = 606	Gut microbiota composition (16S rRNA sequencing)	Overall survival, treatment-related mortality	Identified that lower baseline intestinal microbiota diversity is a significant independent predictor of increased mortality and adverse outcomes post-transplantation [16].
Phillips et al., 2023 [17]	Retrospective cohort study	Adult childhood cancer survivors	*N* = 1413	Modifiable lifestyle risk factors (diet, physical inactivity)	Late-onset cognitive impairment prevalence	Found significant associations between poor modifiable risk factors (including suboptimal diet and sedentary lifestyle) and the emergence of late-onset cognitive impairment [17].
Prasad et al., 2015 [6]	Retrospective cohort study (CCSs cohort)	Adult survivors of adolescent and early young adult cancer	N = 6192 survivors (vs. *N* = 390 siblings)	Exposure to intensive antineoplastic therapy	Psychosocial and neurocognitive outcomes	Established the clinical reality of “sleeper effects,” wherein delayed neurocognitive deficits and psychosocial burdens manifest prominently in early adulthood [6].
Rotz et al., 2022 [3]	Exploratory cross-sectional study	AYA cancer survivors (mixed solid/hematological)	*N* = 35 survivors (vs. *N* = 32 controls)	Fecal microbiota profiling (16S rRNA sequencing)	Metabolic syndrome criteria, systemic inflammatory markers	Demonstrated persistent late-onset dysbiosis; reduced alpha/beta diversity and depleted *Faecalibacterium* correlated with elevated IL-6, CRP, and metabolic syndrome [3].
Shono et al., 2016 [18]	Retrospective clinical analysis	Allogeneic HSCT recipients	*N* = 857 (Total cohort across studies)	Broad-spectrum antibiotic administration	GVHD severity, gut microbiota composition	Broad-spectrum antibiotics severely disrupted commensal flora, which correlated with an increased incidence of severe gastrointestinal GVHD and GVHD-related mortality [18].
Tonning Olsson et al., 2024 [19]	Longitudinal, retrospective cohort study	Pediatric brain tumor survivors	*N* = 199	Oncological treatment protocols (cranial irradiation)	Standardized neurocognitive testing (intelligence quotient, working memory)	Documented progressive IQ decline (up to 20–50 points) and profound attention/working memory deficits exacerbating long-term psychosocial isolation [19].
Zheng et al., 2026 [20]	Retrospective cohort study (CCSs cohort)	Adult survivors of childhood cancer	*N* = 3023	Neurocognitive impairment	Financial hardship, socioeconomic capabilities	Highlighted financial toxicity; neurocognitive deficits hindered patients’ ability to navigate healthcare systems, secondarily burdening the HPA axis through chronic stress [20].

**Table 2:** Long-term cognitive and psychosocial consequences in patients with a history of cancer, broken down by cancer type.

History of Neoplasm Type	Prevalence of Memory and Learning Disorders	Characteristic Psychosocial and Cognitive Complications
Central nervous system tumors	25.4% [14]	Progressive decline in intelligence quotient, profound attention and working memory deficits, low self-esteem, high risk of unemployment and isolation [14].
Leukemias (e.g., acute lymphoblastic leukemia)	13.3% [14]	Executive function disorders manifesting during puberty, reduced capacity to undertake complex tasks associated with methotrexate impact.
Sarcomas (bone and soft tissue)	Below 10% (general symptoms) [11]	Reduced processing speed in 34.5% of patients, decreased cognitive flexibility. Exacerbation of symptoms with low physical activity [14].
Wilms tumor	Below 10% (general symptoms) [1]	Lower scores in verbal fluency and mathematics tests, higher risk of university non-completion. Correlation with secondary nervous system disorders [1].

**Table 3:** Clinical translation of gut–brain axis disruptions into specific neurocognitive and psychosocial symptoms in childhood cancer survivors.

Gut–Brain Axis Communication Pathway	Microbiological or Metabolic Component	Translation to Neurocognitive and Psychosocial Symptoms in CCSs
Neuroimmunological	Bacterial toxins (LPS), cytokines (IL-1β, IL-6) [34].	Disruption of the blood–brain barrier, microglial activation, cortical neuroinflammation, and the phenomenon of brain fog [34].
Metabolomic (SCFAs)	Decreased production of butyrate and propionate resulting from the depletion of *Firmicutes* [34].	Reduced synthesis of brain-derived neurotrophic factor, impaired neurogenesis, and progressive problems with attention and memory consolidation [34].
Afferent pathways and HPA axis	Cortisol production in response to psychological stress in childhood cancer survivors [14,20,34].	Intestinal epithelial damage, selection of pathobionts utilizing cortisol as a growth factor, chronic stress, and anxiety intensification [34].
Tryptophan-Kynurenine pathway	Overgrowth of *Intestinibacterand Megasphaera* species [7].	Shifting the serotonin pathway toward neurotoxic kynurenine, leading to neuronal apoptosis and profound depressive states [7].

**Table 4:** Communication pathways of the gut–brain axis and neurocognitive and psychosocial symptoms in childhood cancer survivors.

Dietary Component and Nutritional Intervention	Gut Microbiome Modulation	Benefits for Neurocognitive and Psychosocial Health of CCSs
Fiber and Cellulose (MIND Diet)	Provision of substrates for massive SCFA production. Stabilization of the *Firmicutes* to *Bacteroidetes* ratio [51,52,53,54].	Restoration of blood–brain barrier integrity, prevention of neuronal apoptosis, and improvement in focus and memory [51,52,53,54].
Fermented Products (Yogurts, Pickles)	Supply of live *Lactobacillus* and *Bifidobacterium* cultures [63,64,65,66].	Redirection of the metabolic pathway toward GABA and serotonin synthesis; alleviation of anxiety and insomnia [63,64,65,66].
Antioxidants and Anthocyanidins (Berries)	Suppression of oxidative stress in the intestinal lumen and protection of beneficial bacterial strains [44].	Abolition of the intracranial neuroinflammation phenomenon, accompanied by direct pro-cognitive and antidepressant effects [5].
Omega-3 Fatty Acids (Marine Fish, Nuts)	Systemic anti-inflammatory action and restriction of pathogen overgrowth [59,60,61,62].	Reconstruction of cell membrane structures in the cortical and hippocampal regions, and alleviation of fatigue syndrome [59,60,61,62].

## Data Availability

No new data were created or analyzed in this study. Data sharing is not applicable to this article.

## Abbreviations

The following abbreviations are used in this manuscript:

ALL	Acute lymphoblastic leukemia
APOEε4	Apolipoprotein E epsilon 4 allele
AYA	Adolescent and young adult
BBB	Blood–brain barrier
BDNF	Brain-derived neurotrophic factor
CCSs	Childhood cancer survivors
CD4+	Cluster of differentiation 4
CD8+	Cluster of differentiation 8
CNS	Central nervous system
DHA	Docosahexaenoic acid
DSM-5	Diagnostic and Statistical Manual of Mental Disorders, Fifth Edition
EPA	Eicosapentaenoic acid
GABA	Gamma-aminobutyric acid
HLA-DR	Human Leukocyte Antigen-DR isotype
HPA	Hypothalamic–pituitary–adrenal
HSCT	Hematopoietic stem cell transplantation
Iba-1	Ionized calcium-binding adapter molecule 1
IL-1β	Interleukin 1 beta
IL-6	Interleukin 6
LPS	Lipopolysaccharide
MeSH	Medical Subject Headings
MIND	Mediterranean-DASH Intervention for Neurodegenerative Delay
NMDA	N-methyl-D-aspartate
PedsQL	Pediatric Quality of Life Inventory
PEO	Population, Exposure, and Outcomes
QoL	Quality of life
RCTs	Randomized controlled trials
SANRA	Scale for the Assessment of Narrative Review Articles
SCFAs	Short-chain fatty acids
TBI	Total body irradiation
TNF-α	Tumor necrosis factor alpha
